# Air Pollution and Its Devastating Effects on the Central Nervous System

**DOI:** 10.3390/healthcare10071170

**Published:** 2022-06-23

**Authors:** Carmen Adella Sîrbu, Ion Stefan, Rodica Dumitru, Marian Mitrica, Aida Mihaela Manole, Titus Mihai Vasile, Constantin Stefani, Aurelian Emil Ranetti

**Affiliations:** 1 Department of Neurology, ‘Dr. Carol Davila’ Central Military Emergency University Hospital, 010242 Bucharest, Romania; sircar13@yahoo.com (C.A.S.); rodica.dumitru95@yahoo.com (R.D.); 2 Department of Infectious Diseases, ‘Dr. Carol Davila’ Central Military Emergency University Hospital, 010242 Bucharest, Romania; 3 Department of Medico-Surgical and Prophylactic Disciplines, Titu Maiorescu University, 031593 Bucharest, Romania; 4 Clinical Neurosciences Department, University of Medicine and Pharmacy “Carol Davila” Bucharest, 050474 Bucharest, Romania; mtvasile@yahoo.com; 5 Department of Neurology, Clinical Ambulatory, ‘Dr. Carol Davila’ Central Military Emergency University Hospital, 010242 Bucharest, Romania; aidamihaelamanole@gmail.com; 6 Department of Family Medicine and Clinical Base, ‘Dr. Carol Davila’ Central Military Emergency University Hospital, 010242 Bucharest, Romania; cristianstefani@yahoo.com; 7 Department No. 5, University of Medicine and Pharmacy “Carol Davila”, 050474 Bucharest, Romania; 8 Department No. 2, University of Medicine and Pharmacy “Carol Davila”, 050474 Bucharest, Romania; ranetti@gmail.com

**Keywords:** stroke, air pollution, neuroinflammation, cardiovascular risk, atrial fibrillation, atherosclerosis

## Abstract

Air pollution is a real public health problem, it being one of the five most common causes of mortality in developing countries. However, pollution studies have focused on the cardiovascular and pulmonary systems in recent decades. Recently, researchers have moved towards a new direction, tracing a direct link between pollution and stroke. Stroke has many known risk factors such as smoking, a sedentary lifestyle, and hypertension. Pollution is universally widespread, already a matter of public interest, so that, although intuitive, it is difficult to connect the two. The particles found in the air that we breathe, regardless of their origin, can attack the body in different ways, causing inflammation, and triggering a true cascade of phenomena that end up attacking the central nervous system and other organs. This article tries to explain the series of phenomena that determine the harmful effect of particles present in the air, with an increased focus on the central nervous system and especially on strokes. A deeper understanding of these phenomena helps in guiding future studies and finding viable solutions to protect people at risk.

## 1. Introduction

Air pollution (AP) is a real public health problem, it being one of the five most common causes of mortality in developing countries [1]. Although the intuitive link between pollution and diseases such as myocardial infarction, diabetes, or other lung pathologies was established, a potential connection between pollution and central nervous system diseases has had increased interest in recent years (Table 1) [2].

Given that stroke is globally the second leading cause of death and the leading cause of disability [3], the identification of all risk factors, including the possibility of prevention and treatment, has become a matter of wide interest, with special importance for public health [4,5].

The usual risk factors involved in ischemic events are numerous, including smoking, an unbalanced diet, excessive alcohol, hypertension, a sedentary lifestyle, and dyslipidemia [6,7]. Beyond all other known risk factors, AP appears to be the third leading cause of stroke in the world, and it is a changeable contributing factor independent of human behavior [8,9]. The World Health Organization (WHO) estimates that AP is responsible for approximately 4.2 million premature deaths annually, accounting for up to 30% of stroke causes in developing countries, and it had more victims in previous years than COVID-19 did [10,11,12]. Moreover, recent studies estimate that, in the event of delays in pollution reduction measures, the number of deaths could double in the next 30 years [13]. During the current pandemic, the initial periods of global lockdown unexpectedly led to a decrease in pollution levels. At the same time, there was a dramatic decrease in the incidence of stroke during the initial period of the COVID-19 pandemic, even though people had a more sedentary lifestyle and an inadequate diet during this period. This may have been due, on the one hand, to reduced addressability in the hospital due to the restrictions imposed by the epidemiological situation and, on the other hand, the decrease in the level of pollution may have resulted in a decrease in the number of vascular events [3,14]. For a long time, the link between pollution and stroke was not universally accepted. First, many studies included both ischemic and hemorrhagic events as “strokes”, without an exact delimitation between the two entities, which have different risk factors. Second, the measurements did not have the necessary accuracy, which could have led to errors. In addition, the pathogenic mechanisms are incompletely known [3]. The present article brings to the fore the main types of polluting particles with their sources of origin, but also their impact on the CNS or the cardiovascular system. Although the exact mechanisms of pollution leading to the degradation of the nervous tissue are incompletely elucidated, this article addresses possible pathophysiological mechanisms, designed to allow for an indepth understanding of the devastating effects of these particles around us.

## 2. Air Pollution and Polluting Particles

AP is a comprehensive term that refers to thousands of harmful particles found in the air of various sources that affect the state of health, and can be found in liquid, solid, or gaseous form, often as mixtures in varying percentages [15]. Particulate matter (PM) represents a mix of extremely heterogeneous substances, regardless of the shape, size, and chemical composition. Increased attention is paid to particles with a diameter of less than 10 µm, because, due to their small size, they can be easily inhaled, later reaching the alveolar level and from there the whole body [16]. The typical classification and recognition of PM are performed according to their size, as follows [15,17,18]. PM10 (particles with a maximal diameter of 10 µm), whose source is represented by the soil industry, mining, or dust produced by vehicles or after construction; and PM2.5 (particles with a maximal diameter of 2.5 µm), resulting from the use of fuels, from the burning of oils or wood, composed of nitrates, sulfates, or complex organic molecules. In addition, substances resulting from the combustion of diesel vehicles participate in the production of PM2.5. A subcategory is represented by PM0.1 (particles with a maximal diameter of 0.1 µm). Particles emitted by burning fossil fuels are a major source of pollution. SO2 is also obtained by burning fossil fuels. Instead, gaseous components come from burning fossil fuels, heavy traffic, and extreme temperatures in some industries [15,19]. Carbon-based fuels used in oxygen-poor atmospheres or at extreme temperatures are CO generators. Taking the above into account, it is easy to deduce that the composition of the air differs in various regions of the globe, taking into account urban or rural areas, the degree of development of various areas, and especially local customs. In developed countries, transport is the main source of pollution, emitting huge levels of PM, along with activities necessary for daily living and the agricultural industry. For example, data released by the UK government estimate that the industry is responsible for producing about half of NO2 and 36% of PM10 emissions, while transport produces about 30% of NO2 and 18% of PM10. In extreme low-income countries, the use of biomass and solids is the main source of pollution, especially in homes. Burning coal or unprocessed wood is a significant source of pollution, given that countries such as South Africa or a significant part of South Asia use these resources to heat or cook in the traditional way [20]. For example, in India, the main sources of air pollution are biomass or coal combustion, used as heat or energy generators, windblown dust, agriculture, the construction industry, and, to a lesser extent, transportation and diesel combustion. Instead, in indoor environments, the burning of fossil fuels such as wood, coal, or residues obtained from daily activities is a major source of pollution. A recent study in India not only strengthens the existing evidence of PA damage, but also demonstrated the improved air parameters during the lockdown imposed by the COVID-19 pandemic. Instead, with the adoption of relaxation measures, pollution levels returned to an upward slope, demonstrating the link between PM production and daily human activity [21]. Pollution varies not only spatially but also temporally and is strongly influenced by various environmental conditions, even depending on the time of day or season. For example, the presence of wind, speed, or direction influences the concentration of PM, temperature contributes intensely to the formation of ozone, so pollution reaches a significantly higher level during the hours of the day with strong sunlight [15,22,23]. On the other hand, recent studies in Ireland suggested a strong association between PM2.5 pollution and the high rate of stroke events in the winter months [24,25]. Given the recent increased interest in pollution levels, many countries are trying to meet the recommended quality standards. Recent studies in the UK show that, in recent decades, PA has declined significantly in many high-income countries, but has tended to stagnate in recent years. On the other hand, in middle- and low-income countries, the situation is constantly deteriorating, and the levels of polluting particles are constantly rising in the USA, and countries in Africa and Asia [20]. Thus, taking into account PM2.5 exposure (µg/m^3^), the most polluted country in the world is Bangladesh (77.1 µg/m^3^), followed by Pakistan (59.0 µg/m^3^) and India (51.9 µg/m^3^). The least polluted countries are Puerto Rico, New Caledonia, and the US Virgin Islands (3.7 µg/m^3^) [26]. Romania has a PM2.5 exposure of 15.8 µg/m^3^ [27]. 

**Table 1:** Main gaseous pollutants and their effects on different systems or organs.

References	Polluting Substance	Source of Production	Effect on the CNS	Other Systemic Effects
W.S. Tunnicliffe et al. (2000) [28]; Nan Sang et al. (2010) [29]; Kuan Ken Lee et al. (2018) [15]	SO2	Released from fossil fuel power plants	Inhaling SO2 could aggravate existing ischemic brain damage	It promotes vasoconstriction in both healthy adults and those with pre-existing asthma
M.A. Go’mez-Garcıa (2004) [30]; Radim J. Sram et al. (2017) [31]; Denise Felber Dietrich et al. (2008) [32].	NOx NO2	Motorized traffic, destruction by incineration of waste, and burning of coal or oil	NO2 is associated with dementia. Nox is associated with Parkinson’s disease.	NO2 exposure is associated with autonomic cardiac dysfunction, especially in women and patients with underlying cardiovascular diseases.
Richard J. Levy (2014) [33]; Cyril Reboul et al. (2017) [34].	CO	Vehicle exhausts, industrial combustion, cigarette smoke, gas cookers, charcoal grills.	Exposure to low levels causes episodes of headache, dizziness, and impaired judgment. Exposure to high levels can cause seizures.	It aggravates myocardial contractile dysfunction, especially in prone patients; CO can cross the placenta, with the possibility of the neurodevelopmental alteration of the fetus
Helen H. Suh et al. (2000) [35]; Liu X. et al. (2022) [36]	O3 (Ozone)	O3 is formed through the interaction between NO2 and volatile organic compounds, under the action of sunlight	Memory impairment, lethargy, severe fatigue, headache episodes, sleep-wake disturbance	Irritating respiratory membranes and the entire respiratory system. Increases the frequency of asthma attacks, weak the immune system, decrease metabolic function

Legend: CNS = central nervous system; SO2 = sulfur dioxide; NOx = nitrogen oxides; NO = nitrous oxide; CO = carbon monoxide; O3 = ozone.

## 3. Effects of AP on Brain Development

However, AP is not only associated with organic abnormalities. Studies conducted in 2010 [37] highlighted the association between elevated levels of PM10, PM2.5, NO2, CO, SO2, and O3, and the occurrence of depression in the elderly population, and emotional instability and suicide attempts among adolescents. In addition, the experimental exposure of laboratory animals to high levels of PM2.5 led to the expression of depressive states and the production of proinflammatory cytokines in the hippocampus, while exposure to nanoparticles in the prenatal period led to behavioral disorders at the age of maturity [38,39].

The impact of PA on brain development in the womb and childhood is not well-known, but studies in recent years have found significant neuroinflammation and oxidative stress in children exposed to high levels of PM. These children have unusual white matter vascularity, perivascular inflammation, and blood–brain barrier (BBB) impairment, which explains the detection of autoantibodies to the brain occludin (an enzyme that oxidizes nicotinamide adenine dinucleotide, a coenzyme with a central role in the cell metabolism) and zonulin (a protein that modulates the permeability of tight junctions between cells). The data collected from these studies conclude that children living in highly polluted environments show that such mechanisms lead to the alteration of CNS tissue, with a predisposition to the further development of Alzheimer’s disease and Parkinson’s disease [38]. 

## 4. Stroke and Its Connection to Pollution

A stroke is defined as an episode of ischemic or hemorrhagic neurological disability that persists for at least 24 h. It is a significant cause of mortality and morbidity, and is found in higher percentages in populations of low–middle income countries, but with a decreasing incidence in developed countries. Stroke is classified into two major categories: ischemic, usually caused by a thrombus or embolus that occludes a certain artery with alteration of the tissue served by the vessel; and hemorrhagic, parenchymatous, or subarachnoid, generally due to the rupture of an underlying vessel or aneurysm [14,20]. Although patients with cerebral vascular events have many common risk factors, such as dyslipidemia, a sedentary lifestyle, smoking, and hypertension, there is growing evidence for pollution contributing as a risk factor for stroke, depending on the duration of the exposure to polluted particles [14,15,40]. Thus, when we talk about the link between vascular events and pollution, we can refer to two directions: short-term and chronic exposure.

### 4.1. Short-Term Exposure

The link between pollution and the onset of cerebrovascular disease began to be established almost 40 years ago when several studies in China raised the suspicion that smoke emitted inside homes after burning coal is an important risk factor for stroke regardless of other parameters such as blood pressure or smoking [41,42]. Numerous recent studies, including systematic reviews or meta-analyses, suggest that exposure to high concentrations of PM2.5 and PM10 would be associated with an increased risk of stroke morbidity and mortality, with stronger evidence for PM2.5 [1,15,25]. Sometimes, a sudden increase in the level of pollution, such as spikes found during some days, can result in vascular events, such as the rupture of an atheromatous plaque causing an ischemic stroke or the rupture of an aneurysm causing a hemorrhagic stroke. Acute effects of pollution also include the increased procoagulant status of the blood or increased blood pressure, events that can also lead to the sudden onset of a stroke [14,20]. 

### 4.2. Long-Term Exposure

Long-term impairment refers to the results of exposure to elevated PM levels over months or years. Studies in this direction suggest that the adverse effects are mainly due to exposure to PM2.5, which is associated with an increased incidence of cardiovascular disease but also increased mortality, especially among postmenopausal women [43,44,45,46]. In addition, long-term environmental pollution has been associated with accelerated atherosclerosis and increased vulnerability for atheroma plaques, with the pathological substrate being the thickening of the intima and carotid arteries. Carotid artery stenosis, a well-known etiological factor for stroke, has recently been independently associated by the latest studies with pollution after adjusting the cardiovascular parameters [15,47,48,49]. In addition to carotid artery stenosis, studies mostly conducted on the harmful effects of PM 2.5 suggest other harmful effects of air pollutants such as the thickening of the intima and media carotid artery, and the calcification of coronary arteries, the thoracic and abdominal aorta [20,50]. AP is the most important environmental risk factor for mortality from any cause, so many studies have been conducted to bring more information in this direction, as there is already a link between stroke and various types of PM, but data on vascular events and gaseous pollutants were inconclusive. Thus, a recent study conducted by Z. Niu et al. [4] highlighted the strong link between them and hospital admission incidence, and mortality.

## 5. Pathophysiological Mechanisms

The real danger of these pollutants is that the filters of the nasal and pulmonary passages are not effective in retaining these fine particles, which have a high capacity to penetrate the parenchyma, with consecutive passage into the bloodstream and then into the whole body [17,51]. The mechanisms that may explain the sudden onset of these vascular events are thrombosis, vasoconstriction, the rupture of unstable atheromatous plaques, or sudden changes in blood pressure.

### 5.1. Eternal Theory of “Inflammation”

Once they enter the body, these fine materials are deposited into the lung parenchyma, where the local defence cells, macrophages, come into action, which triggers the local immune response, with subsequent systemic spread and endothelial dysfunction [9,52,53]. Studies in this direction have identified elevated levels of circulating cytokines in individuals exposed to elevated PM levels, such as interleukin types 1 and 6, tumor necrosis factor, and biologically inflammatory syndrome. These changes trigger an entire biological cascade, generating a general inflammatory status, namely, oxidative stress, which amplifies the harmful effect of pollutants and maintains this vicious circle. In addition, in the serum of patients exposed to AP, there are elevated levels of phospholipase A2, which is considered to be a stand-alone risk factor for stroke [15,40,54]. Short-term exposure to PM2.5 also increases matrix metalloproteinases, which are markers of atheroma plaque vulnerability, but also increase systemic inflammation and thrombogenicity [3,43]. These mechanisms have a marked effect on patients who have a pre-existing heart disease, and may cause a sudden ischemic vascular event of an atherothrombotic nature.

### 5.2. Nanoparticle Translocation Theory

Before the mentioned cytokines can activate following their circuit through the circulatory stream, endothelial cells are found in the cerebral vascularization with the subsequent alteration of the BBB. The alteration of this brain protection system can lead to the translocation of various inflammatory markers in the brain parenchyma, with the production of increased amounts of CD163 or CD68, which are inflammatory markers expressed by inflammatory cells present at that level that maintain a proinflammatory status in the brain. The analysis of brain tissue samples obtained from patients living in heavily polluted areas shows their infiltration with numerous infiltrating monocytes or activated microglia, and elevated levels of interleukins, leading to the maintenance of prefrontal lobe lesions [17,55]. Particles emitted by diesel combustion may increase endothelial permeability, so the theory of translocating ultrafine particles has been launched. There is evidence that confirms the possibility of PM crossing the olfactory mucosa, tissue in direct contact with the outside environment, but especially with air rich in particles. Thus, the pollen deposited at this level can penetrate the mucosa through a simple diffusion mechanism called pinocytosis, reaching receptor neurons at that level. Later, it is transported from close to the axonal level to the olfactory bulb and then to the central level, affecting various brain structures in the vicinity [15,17].

### 5.3. Autonomic Dysfunction

As previously explained, inhaled particles, once deposited in the lung parenchyma, cause local inflammation, with the activation of neural sensory receptors, which in turn causes autonomic dysfunction with impaired normal cardiovascular function. Patients exposed to PM2.5 have impaired cardiac activity and a predisposition to atrial fibrillation, an etiological factor established for stroke [15,56]. Another theory that may underlie vascular events is the alteration of the reflex airway. It appears that the pollutants may interact with pulmonary chemoreceptors and baroreceptors, inducing autonomic dysfunction that results in increased vascular resistance, arrhythmias, and hypertension. All of this can result in a vascular event of an ischemic nature, a cardioembolic etiology, or a hemorrhagic nature [3].

## 6. What Can We Do?

The above shows that AP is a real public health problem, and despite growing and compelling evidence that it contributes heavily to stroke mortality and morbidity, many states do not meet the recommended air quality criteria. The ongoing studies are intended to raise the alarm and prompt the competent authorities to take the necessary measures to reduce the effects. Thus, to reduce PM pollution, it is recommended to reduce or even give up the use of conventional fuels and reorient to more environmentally friendly sources, which would not only reduce the harmful effects on the body but also reduce drastic climate change. In terms of transportation, it is recommended that measures be taken against vehicles with high PM emissions, and residences be constructed away from the crowded heavily trafficked areas of large cities. Several measures could also be taken at the individual level: it is recommended to use mainly public transport or minimally polluting means (such as bicycles). In addition, limiting going outside during busy hours or in busy areas could reduce the harmful effects of PA [15,57,58,59]. A series of portable sensors capable of detecting individual metabolic activity, heart rate, respiratory rate, and the number of steps performed daily have become readily available globally. These devices should be used to identify at-risk individuals who require a series of preventive measures, such as avoiding congested areas, especially during peak hours, with heavy traffic [3,60]. The well-being of our planet as a comprehensive universal environment for the entire population should become a priority for each person. In the Philippines, a series of educational programs have been launched that focus on the problems of the planet, accompanied by the launch of beneficial ideas and solutions to minimize pollution damage and maximize planetary health. These programs have been implemented in several institutions, such as St. Luke’s College of Medicine, and the Microbiology for Health and Environment Research Group at the University of the Philippines Manila, and their introduction as a subject in the school curriculum is under discussion [61]. The current discoveries and associations are according to studies based on measurements for outdoor environments based on fixed measuring stations, so that future studies require the extension of research for both outdoor and indoor air, and a variability in values depending on the various daily activities and areas [20].

## 7. Conclusions

Although the effects of AP on the respiratory tract were already known, recent studies reveal its devastating effects on the central nervous system as well, but the underlying mechanisms are complex and still incompletely elucidated. Neurological diseases are the second leading cause of disability after cardiovascular disease, so risk factors for CNS require increased attention, which should be a future research direction. Despite human evolution and the increasingly aggressive popularization of alternative energy sources, the burning of fossil fuels continues to massively affect the quality of the air that we breathe and hinder moving towards a greener planet.

## Data Availability

Not applicable.
